# Nonscrapable yeast: A case of chronic hyperplastic candidiasis following secukinumab therapy

**DOI:** 10.1016/j.jdcr.2025.06.025

**Published:** 2025-06-30

**Authors:** Gabriela A. Duchesne, Renee M. Copeland, Rafik A. Abdelsayed, Loretta S. Davis

**Affiliations:** a Medical College of Georgia at Augusta University, Augusta, Georgia; b Department of Dermatology, Medical College of Georgia at Augusta University, Augusta, Georgia; c Department of Oral Biology and Maxillofacial Pathology, Dental College of Georgia at Augusta University, Augusta, Georgia

**Keywords:** chronic hyperplastic candidiasis, IL-17 inhibitors, oral candidiasis, secukinumab

## Introduction

Oral candidiasis is the most common opportunistic oral infection, typically presenting as easily scrapable, white mucosal plaques or papules known as pseudomembranous candidiasis. In contrast, chronic hyperplastic candidiasis (CHC), a rare variant occurring in approximately 0.04% to 1.61% of cases, presents as nonscrapable, adherent white plaques, at times referred to as candidal leukoplakia.^1^, ^2^, ^3^ We present a case of CHC in a patient with several risk factors, specifically diabetes mellitus and Behçet disease, treated with chronic immunosuppressive medications including interleukin 17 (IL-17) inhibitor therapy.

## Case report

A 51-year-old Asian man with type 2 diabetes mellitus, herpes simplex virus type 1 on suppressive valacyclovir therapy, and Behçet disease managed with azathioprine, prednisone, apremilast, colchicine, and secukinumab was referred for dermatologic evaluation. He presented with concerns of episodic, painful oral outbreaks of his buccal mucosa, palate, and tongue, which developed approximately 1 year after his Behçet disease diagnosis was stabilized with the addition of secukinumab. Secukinumab was discontinued by rheumatology shortly before his visit to dermatology.

**Fig 1:**
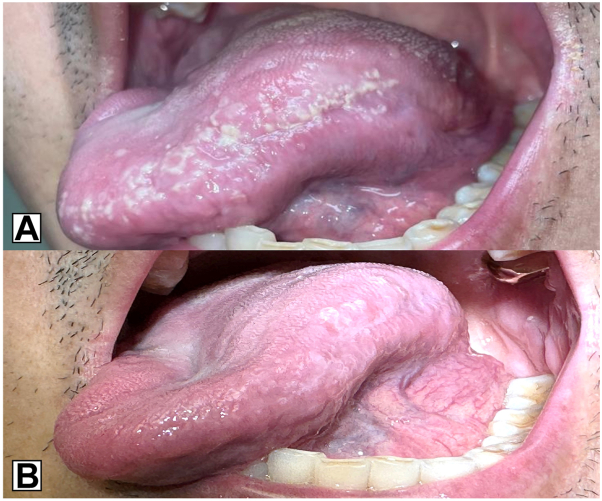
**A,** White punctate papules and gray-white plaques cover the left lateral tongue. **B,** Nystatin therapy and discontinuation of secukinumab resulted in significant improvement with minimal residual disease at postbiopsy follow-up.

**Fig 2:**
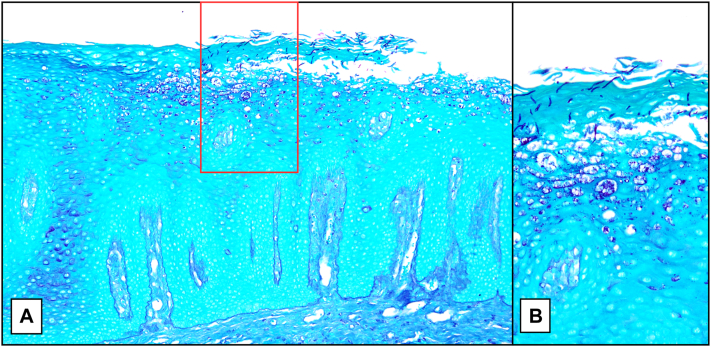
**A,** Periodic acid-Schiff stain demonstrates hyperkeratosis with acanthosis. **B,** Numerous Candida pseudohyphae, many lying perpendicular within the stratum corneum.

Examination of the oral cavity revealed nonscrapable white punctate papules forming gray-white plaques along the lateral margins of the tongue (Fig 1, *A*). Differential diagnosis included a lichenoid reaction and candidiasis, which was considered less likely, given the nonscrapable clinical findings. herpes simplex virus type-1 polymerase chain reaction was negative. He was referred to Oral and Maxillofacial Pathology where he underwent a punch biopsy. Histopathologic examination revealed a hyperplastic mucosal epithelium surfaced by a thick shaggy parakeratin, supporting numerous aggregates of neutrophils with acanthosis of the spinous cell layer. The lamina propria supported superficial and deep mixed and diffuse inflammatory cell infiltrates. Periodic acid-Schiff stain revealed numerous yeast forms and pseudohyphae of *Candida*, many lying perpendicularly within the keratin layer, confirming the diagnosis of CHC (Fig 2). Epithelial dysplasia was not present in the examined tissue sections. Discontinuation of secukinumab led to initial improvement, with complete resolution following treatment with oral nystatin therapy (Fig 1, *B*).

## Discussion

CHC is a rare variant of oral candidiasis presenting with nonscrapable white plaques or speckled lesions that most commonly affect the dorsal and lateral tongue as well as the oral commissures of the buccal mucosa.^1^ CHC most commonly affects men older than the age of 50 years with risk factors including immunosuppression, tobacco use, dentures, diabetes mellitus, nutritional deficiencies, trauma, and hyposalivation.^1^^,^^3^^,^^4^

The underlying pathogenesis of CHC remains poorly understood. It is thought to occur secondary to either *Candida*-induced hyperkeratosis or as a result of leukoplakia with superimposed *Candida*.^1^^,^^4^ *Candida*’s ability to undergo phenotype switching and use hyphal appendages for tissue invasion enhances its survival in disease states and promotes epithelial penetration in CHC.^3^

If Candida mucositis is suspected and scraping does not yield a specimen for potassium hydroxide examination, a biopsy should be performed. Definitive diagnosis of CHC requires histologic examination with periodic acid-Schiff staining to confirm the presence of candidal colonization and assess for CHC-associated dysplasia.^4^ Although epithelial colonization with *Candida* is observed in biopsies of pseudomembranous candidiasis,^5^ the histopathology of CHC and pseudomembranous candidiasis varies significantly. CHC classically demonstrates epithelial hyperplasia with hyperparakeratosis, microabscesses composed of polymorphonuclear leukocytes, and epithelial hyphae lying perpendicular to the stratum corneum.^4^ In contrast, pseudomembranous candidiasis demonstrates intraepithelial and subepithelial inflammatory cells, microabscesses, and tangles of numerous superficial hyphae, clinically “scrapable.”^5^

CHC has been considered a risk factor for the development of squamous cell carcinoma. In 1 study of 48 patients with CHC, 10 had associated epithelial dysplasia, and 2 experienced malignant transformations.^4^ However, the potential role of candidiasis in the pathogenesis of epithelial dysplasia is controversial.^1^ A 2022 analysis of existing literature concluded that the association between *Candida* and epithelial dysplasia is coincidental and that mucosal alterations create an opportunistic environment for fungal dysbiosis.^6^ However, further research to investigate the association was recommended. Notably, it is not uncommon that candidal leukoplakia demonstrates reactive epithelial alterations.^1^^,^^4^^,^^6^

Biopsy remains the current standard for diagnosis of CHC. Empiric antifungal therapy for suspected CHC is controversial, especially since epithelial changes may persist for a period of time post-treatment, complicating the management of patients without a confirmed diagnosis.^7^

Given the uncertainty regarding the exact biologic nature of CHC and the possibility of dysplastic lesions superimposed with candidiasis, prompt treatment with antifungals such as nystatin oral suspension or oral fluconazole and timely follow-up are essential to ensure resolution. One study observed that treatment with oral fluconazole resulted in a lower rate of recurrence compared with oral nystatin rinsing.^4^ CHC lesions that persist following antifungal therapy should be rebiopsied.

The IL-17 pathway plays a critical role in defending against fungal infections, including *Candida*. IL-17 inhibitors, such as secukinumab, are Food and Drug Administration-approved for multiple inflammatory conditions, including psoriasis, psoriatic arthritis, and hidradenitis suppurativa. They are known to increase susceptibility to candidiasis.^8^, ^9^, ^10^ One study, involving 592 psoriasis patients on biologic therapy, found that fungal infections were more frequently observed in those treated with IL-17 inhibitors. Among the 190 patients treated with IL-17 inhibitors, superficial candidiasis was observed in approximately 8% of the cases, although the specific type of candidiasis was not detailed.^10^

Two prior reported cases, both in the oral medicine literature, link secukinumab to CHC; both resolved with antifungal therapy and did not require discontinuation of secukinumab therapy.^8^^,^^9^ Although the causation of CHC in this case is likely multifactorial, including diabetes and immunosuppression with prednisone and azathioprine, the timing of symptom development, partial improvement following discontinuation of therapy, and the drug’s known risk of Candida infections support an association with secukinumab. In retrospect, oral antifungal therapy may have proved adequate to treat CHC in this case and allowed for the continuation of therapy.

This case underscores the need to include CHC in the differential diagnosis when evaluating nonscrapable oral lesions, particularly in immunocompromised patients. Mucosal biopsy is required for diagnosis and for the evaluation of any associated dysplasia. Candida hyphae positioned vertically within the stratum corneum, nicely demonstrated in this case, is an unusual characteristic of CHC. This vignette serves to highlight a rare yet potentially significant adverse event that may be linked to IL-17 inhibitors. Further research is required to better understand the association between IL-17 inhibitor therapy and CHC.

## Conflicts of interest

None disclosed.
